# A 5-Year Study of Lithium and Valproic Acid Drug Monitoring in Patients with Bipolar Disorders in an Italian Clinical Center

**DOI:** 10.3390/ph15010105

**Published:** 2022-01-17

**Authors:** Marco Carli, Eleonora Risaliti, Mena Francomano, Shivakumar Kolachalam, Biancamaria Longoni, Guido Bocci, Roberto Maggio, Marco Scarselli

**Affiliations:** 1 Department of Translational Research and New Technologies in Medicine and Surgery, University of Pisa, 56126 Pisa, Italy; carlimarco@outlook.it (M.C.); e.risaliti@studenti.unipi.it (E.R.); m.francomano@studenti.unipi.it (M.F.); k.shivakumar@alumni.sssup.it (S.K.); biancamaria.longoni@unipi.it (B.L.); 2 Department of Clinical and Experimental Medicine, University of Pisa, 56126 Pisa, Italy; guido.bocci@unipi.it; 3 Department of Biotechnological and Applied Clinical Sciences, University of L’Aquila, 67100 L’Aquila, Italy; roberto.maggio@univaq.it

**Keywords:** lithium, valproic acid, therapeutic drug monitoring, subtherapeutic dose

## Abstract

Therapeutic drug monitoring (TDM) is an effective tool used to improve the pharmacological treatment in clinical practice, especially to detect subtherapeutic drug plasma concentration (Cp) in order to consider a change of dosage during treatment and reach its putative therapeutic range. In this study, we report the Cp values of lithium and valproic acid (VPA), alone and in combination, mostly in bipolar patients admitted to an Italian clinical center of the University of Pisa during the years 2016–2020, which include 12,294 samples of VPA, 7449 of lithium and 1118 of both in combination. Lithium and VPA are the most utilized drugs in treating bipolar disorders, and their TDM is strongly recommended by recent guidelines. In relation to lithium Cp monitoring, several studies have underlined that 0.5–0.8 mmol/L is the optimal range for chronic treatment, and below 0.4 mmol/L, it is unlikely to produce a clinical response. For VPA, the therapeutic range is 50–100 μg/mL and a linear correlation between Cp and clinical efficacy has been proposed, where below 50 μg/mL, the clinical efficacy of VPA has not been proven thus far. Toxic levels of both drugs were rarely found in our study, while a high percentage of patients, about one-third, had sub-therapeutic Cp during their treatments. In addition, in several cases of patients receiving multiple blood sampling, the initial subtherapeutic Cp changed only partially without reaching its therapeutic window. In relation to age, we found a higher percentage of lithium and VPA Cp values in range in the adolescents than in the adults and elderly groups. No differences were reported when analyzing the distribution of Cp values in males and females. In conclusion, this present study suggests that TDM is widely used by many specialists, but there is still a window of improvement for optimizing pharmacological treatments in clinical practice.

## 1. Introduction

In psychiatric disorders, about one-third of patients do not show significant improvements from the pharmacological treatments, and one reason of this lack of success is that several patients suspend drug usage during treatment or use medications incorrectly [1].

In addition, since patients can respond differently to the same dose of the same drug, the therapeutic response is often difficult to predict, underling an urgent need for objective references and/or biomarkers while introducing patients to a new medication.

Among factors influencing the pharmacologic response, individual pharmacokinetic characteristics can be responsible for the lack of therapeutic efficacy. Interindividual differences in drug metabolism (e.g., extensive versus poor metabolizers) and drug–drug interactions are issues that need to be considered [2,3,4].

For these issues, therapeutic drug monitoring (TDM) by measuring plasma drug concentration (Cp) is an effective tool to improve current pharmacological therapies, as it provides a therapeutic range for the best probability of therapeutic response combined with reduced risk of adverse drug reactions and toxicity [5]. In addition, it allows for the controlling of patient’s compliance to pharmacological treatments, an aspect that needs not be underestimated. Recent guidelines also recommend the use of TDM for personalized pharmacotherapy, including when switching from one medication to another [6,7].

TDM was initially introduced in psychiatric disorders to avoid toxic levels during chronic use of drugs, such us lithium, whose monitoring is currently mandatory for safety reason.

Later on, a relationship between drug monitoring and better clinical outcome has been proposed for anticonvulsant drugs [6], tricyclic antidepressants [8], antipsychotics [9] and mood stabilizers [10]. For example, for antiepileptic drugs, subtherapeutic levels were found in many patients attending hospitals due to seizures [11]. 

According to a recent survey proposed on 225 psychiatrists in relation to lithium TDM, it emerged how doctors still pay more attention to try to avoid toxicity values rather than reaching Cp levels within the therapeutic range. Only 5% of doctors reported to change the dose when the Cp was below the therapeutic range [7].

Among the several psychiatric diseases, bipolar disorders (BDs) are considered the most severe and complex, usually requiring chronic treatments. BDs are often associated with other psychiatric comorbidities, substance use disorder and a high risk of suicide. In addition, bipolar patients are difficult to manage for their tendency to discontinue pharmacological treatments, especially in the manic state and for their low compliance [12,13]. 

Since its approval in the 1970s, lithium is still considered the gold standard for BDs treatment, especially to prevent a manic episode. Recently, the BALANCE study based on a randomized clinical trial has confirmed lithium monotherapy superiority over VPA and other treatments for relapse prevention in bipolar disorder type I (BD-I) [14].

However, conditions such as mixed states, rapid cycling, and anxiety and substance use disorders comorbidities limit the efficacy of lithium. In these cases, the use of VPA alone or in association with lithium is considered a valid alternative [15,16]. Polypharmacy is commonly used in the treatment of BDs, especially to remit from acute maniac phase [17], and drugs such as atypical antipsychotics are frequently used [18,19,20,21].

In relation to lithium Cp monitoring, several studies have underlined that 0.5–0.8 mmol/L is the optimal serum concentration for chronic treatment. Lithium levels below 0.4 mmol/L are not associated with a clinical response [22]. Similar evidence was found in a study conducted by the University of Pisa [23] on hospitalized patients treated with lithium alone or in association with VPA and carbamazepine. During the observation period, patients with lithium concentrations above 0.50 mmol/L showed significant clinical improvement. Most common adverse reactions of lithium are polyuria/polydipsia, nausea, diarrhea, tremor, cognitive dullness, renal dysfunction and hypothyroidism. For many of them, a correlation with lithium Cp has been described [24,25].

To the best of our knowledge, only one study has demonstrated the possibility to reduce the therapeutic range for lithium to 0.4/0.8 mmol/L when it is administrated together with VPA during the euthymic phase, but not below 0.4 mmol/L [26].

TDM is also strongly recommended for VPA, considering the good relationship between Cp, clinical efficacy, and the onset of toxic effects. The therapeutic window of VPA is 50–100 μg/mL, with an alert threshold of 120 μg/mL. VPA is highly bound to plasma proteins (87–95%) and it inhibits CYP2C9 and glucuronyl transferase, and hence may interact with other drugs. The major routes of VPA metabolism are the glucuronidation and β-oxidation [27]. Some recent evidence suggests that VPA might also mildly induce UGT1A1 when prescribed at high doses [28].

Surprisingly, not many studies analyzed VPA plasma monitoring. In one study, the best clinical response during the acute manic phase was for plasma levels above 90 μg/mL, while in the maintenance treatment, the range between 75 and 99 μg/mL was observed to be optimal for preventing relapse and drug discontinuation. As for lithium, a linear correlation between the plasma concentration of VPA and clinical efficacy has been proposed [29]. Common adverse reactions of VPA include nausea, vomiting, diarrhea, drowsiness, weight gain and tremor, and many of them are dose related [30]. Moreover, rare but life threatening, adverse drug reactions are hepatoxicity and thrombocytopenia [31].

With these premises, in this study, we decided to report the Cp of lithium and VPA, alone and in combination, mostly in bipolar patients admitted to an Italian clinical center of the University of Pisa during the years 2016–2020, including about 12,294 samples of VPA, 7449 of lithium, and 1118 of the two in combination. This analysis has taken into considerations the relevance of gender, age and other parameters to examine their influence on Cp values. 

## 2. Results

### 2.1. Description of the Lithium and VPA Blood Samples during the Period 2016–2020

**Figure 1:**
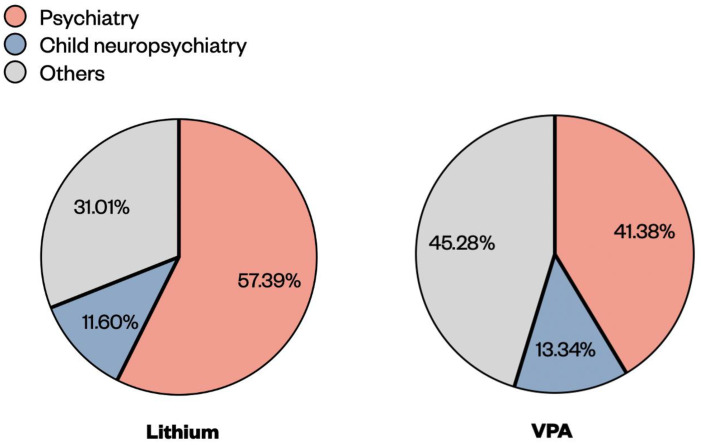
Percentages of lithium and VPA blood samples coming from different clinical units (psychiatry, child neuropsychiatry and others) of the Hospital of the University of Pisa (Azienda Ospedaliero-Universitaria Pisana, AOUP) for the years 2016–2020.

We collected Cp of lithium and VPA from blood samples for a 5-year period from 2016–2020 from the unit of Pharmacology and Pharmacogenetics of the Hospital of University of Pisa (Azienda Ospedaliero-Universitaria Pisana, AOUP). The total number of lithium samples was 7449, where 4275 (57.39%) came from the psychiatry unit, 864 (11.60%) from the child neuropsychiatry unit and the remaining 2310 (31.01%) from other units (Figure 1). For 2016, the number of lithium samples was low because the TDM service had only started since the 1 November. The median values of lithium Cp samples were stable across the years, with a Cp around 0.5 mmol/L (Table 1).

During the same period, a total of 12,294 VPA samples was registered, where 5087 (41.38%) came from the psychiatry unit, 1640 (13.34%) from child neuropsychiatry and the remaining 5567 (45.28%) came from other units (Figure 1). The median values of VPA Cp samples were stable across the years, with a Cp close to 50 μg/mL (Table 1). We noticed that during 2020, the year when the COVID-19 pandemic spread across Italy and demanded several restrictions, the numbers of lithium and VPA samples decreased by 15–20% compared to other years.

### 2.2. Distribution of the Lithium and VPA Cp Samples

**Figure 2:**
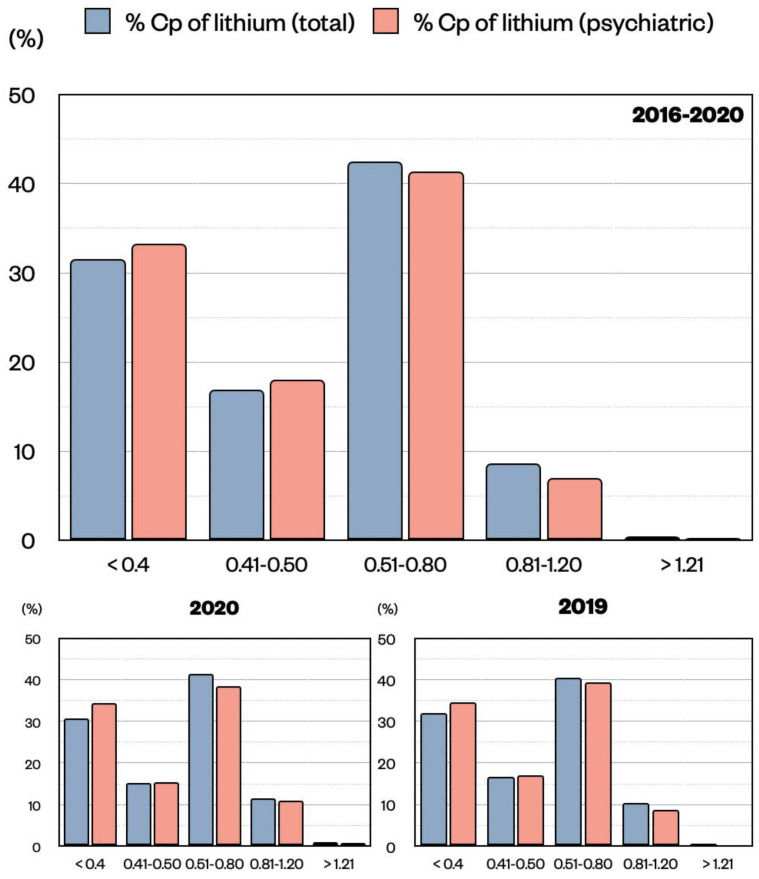
Distribution of lithium Cp (mmol/L) expressed as percentage of the total population (pink) or of the psychiatric unit only (blue) divided by ranges (<0.4, 0.41–0.50, 0.51–0.80, 0.81–1.20, >1.20). Data are shown for all five years 2016–2020, and for the single years 2020 and 2019, respectively. No significant difference was observed in the distribution of Cp samples in the two populations by means of z-test.

Lithium Cp distribution was described by comparing the blood samples coming from all units (total) with the ones coming from the psychiatry unit only. Generally, lithium is used in the psychiatric unit to treat BDs, while in the other units, it can also be used for other neuropathologies (e.g., conduct disorder in child neuropsychiatry). For lithium, the therapeutic window is between 0.5 and 0.8 mmol/L for chronic treatment, and it can be increased up to 1.2 mmol/L during the acute phase. In addition, we decided to highlight the range 0.4–0.5 mmol/L as close to the optimal level, and Cp values < 0.4 mmol/L were considered as subtherapeutic. In reference to lithium total samples, 31.52% were < 0.4 mmol/L, 16.89% between 0.41–0.5 mmol/L, 42.49% between 0.51–0.80 mmol/L, 8.66% between 0.81–1.20 mmol/L, and 0.44% were >1.20 mmol/L (Figure 2). A similar distribution was found for lithium samples from the psychiatry unit alone with 33.29% < 0.4 mmol/L, 18.01% between 0.41–0.5 mmol/L, 41.38% between 0.51–0.80 mmol/L, 7.02% between 0.81–1.20 mmol/L, and 0.30% were >1.20 mmol/L (Figure 2). It is reasonable to think that the vast majority of lithium samples coming from the other units refer to patients suffering from BDs, as it is for the psychiatric unit. In both scenarios, it is clear that about 42% of the samples are in the therapeutic range (0.5–0.8 mmol/L), while the remaining are not, with more than 30% below 0.4 mmol/L. Importantly, values in the toxic ranges are rare. For 2020, the year of the COVID-19 pandemic, we compared the Cp distributions of 2019 and 2020 and found them to be similar (Figure 2). This confirms stable values for lithium across all years.

**Figure 3:**
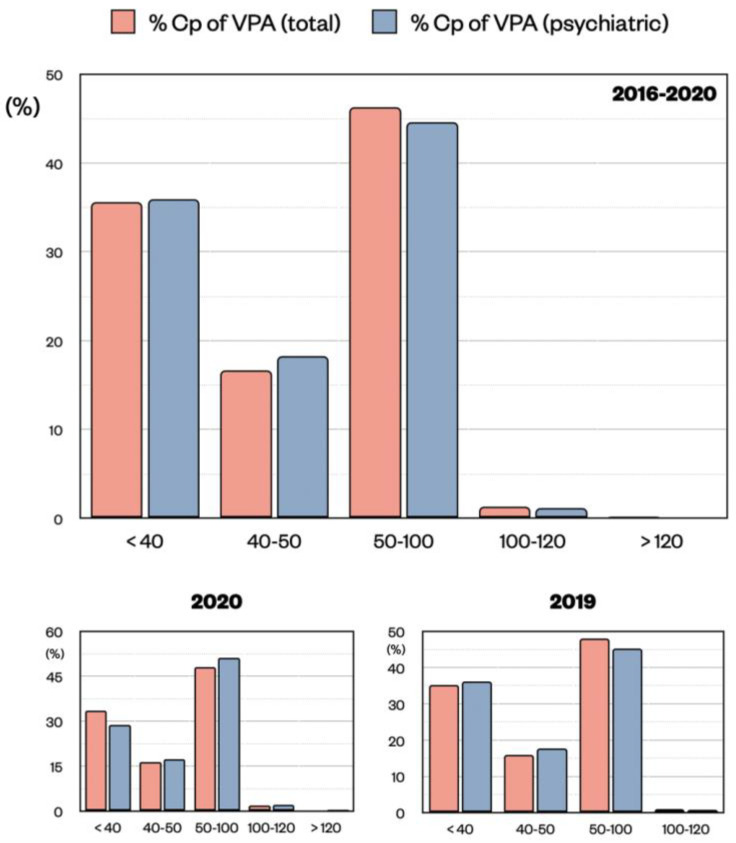
Distribution of VPA Cp (μg/mL) expressed as percentage of the total population (pink) or of the psychiatric unit only (blue) divided by ranges (<40, 40–50, 50–100, 100–120, >120). Data are shown for all five years from 2016 to 2020, and for the single years 2020 and 2019, respectively. No significant difference was observed in the distribution of Cp samples in the two populations by means of z test.

Regarding VPA Cp distribution, we used a similar approach by comparing all blood samples with the ones coming from the psychiatry unit only. Generally, VPA is used in the psychiatric unit to treat BDs, while in the other units, it can also be used for other neuropathologies (e.g., for epilepsy). For VPA, the therapeutic window is between 50 and 100 μg/mL, and the Cp above 120 μg/mL is evaluated as toxic. In addition, we decided to highlight the range 40–50 μg/mL as close to the optimal level and we considered Cp values < 40 μg/mL as sub-therapeutical. In total, 35.64% of the VPA samples were < 40 μg/mL, 16.65% between 40–50 μg/mL, 46.27% between 50–100 μg/mL, 1.29% between 100–120 μg/mL, and 0.16% were >120 μg/mL (Figure 3). A similar distribution was found for VPA samples from the psychiatry unit with 35.39% < 40 μg/mL, 18.22% between 40–50 μg/mL, 44.56% between 50–100 μg/mL, 1.14% between 100–120 μg/mL, and 0.16% were >120 μg/mL (Figure 3). Regarding VPA total samples, one should expect that VPA samples coming from other units (45.28%) could refer to patients suffering from BDs or epilepsy. However, as mentioned above, a similar VPA Cp distribution was found in both medical conditions.

Similar to lithium, almost 50% of the VPA samples are in the therapeutic range (50–100 μg/mL), while the remaining are not, with 35% below 40 μg/mL. Values in the toxic ranges are practically absent. The distributions of 2019 and 2020 (COVID-19) were similar, indicating stable values for VPA across all years (Figure 3).

### 2.3. Distribution of the Lithium and VPA Cp Samples across Different Ages

Since we did not find any particular difference between the totality of samples and the psychiatric ones, we analyzed the distribution of patients’ age and the Cp considering the whole sample during the 5 years.

**Figure 4:**
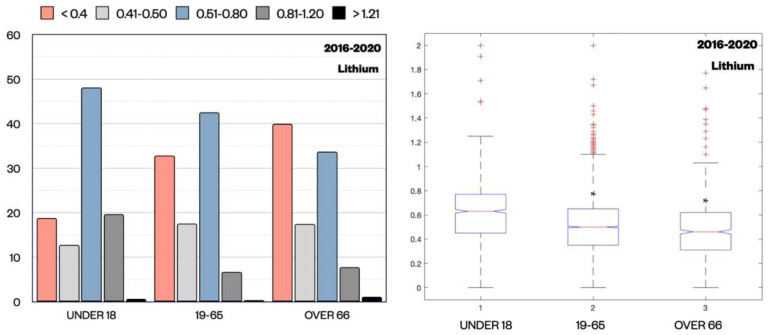
Distribution of lithium Cp (mmol/L) expressed as percentage for each subpopulation divided by ranges (<0.4, 0.41–0.50, 0.51–0.80, 0.81–1.20, >1.20) and by age groups (under 18, 19–65, over 66). Z-test revealed a statistically significant difference in Cp distribution proportions across age groups, in particular for samples < 0.4 mmol/L and between 0.5 and 0.8 mmol/L. The median value of lithium Cp (mmol/L) for each age group is also shown. * *p* < 0.001 compared to the group under 18 by Kruskal–Wallis test (+ outliers samples are marked).

In relation to lithium Cp, we observed that the percentage of samples in the therapeutic range, between 0.5–0.8 mmol/L and 0.8–1.2 mmol/L, was significantly higher for patients under 18 (48.15% and 19.66%) compared to patients aged 19–65 (42.57% and 6.73%) (*p* = 0.024 and <0.001, z-test) and patients over 66 (33.70% and 7.78%) (*p* < 0.001 and 0.056, z-test). In parallel, subtherapeutic Cp (<0.4 mmol/L) was clearly more represented in patients over 66 (39.97%) and in patients aged 19–65 (32.79%), while in patients under 18, it was only in 18.80% of samples (*p* < 0.001, z-test) (Figure 4). This difference in relation to age was also clear in the lithium median values (interquartile range (IQR) is reported in parenthesis) in these three different cohorts, where it was 0.63 (0.32) mmol/L in patients under 18, 0.50 (0.30) mmol/L in patients aged 19–65, and 0.47 (0.31) mmol/L in patients over 66 (*p* < 0.001, Kruskal-Wallis test). These data indicate that in our study lithium Cp values in the adolescents group were more in the therapeutic range compared to the other categories, while in the elderly group, they were the lowest.

**Figure 5:**
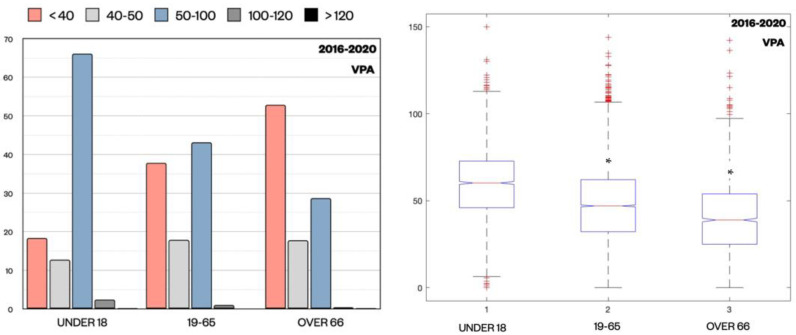
Distribution of VPA Cp (μg/mL) expressed as percentage for each subpopulation divided by ranges (<40, 40–50, 50–100, 100–120, >120) and by age groups (under 18, 19–65, over 66). z-test revealed a statistically significant difference in Cp distribution proportions across the age groups, in particular for samples < 40 μg/mL and between 50 μg/mL and 100 μg/mL. The median value of VPA (μg/mL) for each age group is also shown. * *p* < 0.001 compared to the group under 18 by Kruskal–Wallis test. (+ outliers samples are marked).

Regarding VPA levels distribution, a similar trend to lithium was observed. In the whole sample, 66.14% of patients under 18 were in the therapeutic range (50–100 μg/mL), while only 43.12% of patients aged 19–65 and 28.67% of patients over 66 exhibited values in this range (*p* < 0.001, z-test) (Figure 5).

Again, subtherapeutic levels (<40 μg/mL) were more common in older patients over 66 (52.90%), compared to patients aged 19–65 (37.86%) and in adolescents under 18 (18.38%) (*p* < 0.001, z-test). The VPA median values (interquartile range (IQR) is reported in parenthesis) among these three different cohorts were 60 (17.95) μg/mL in patients under 18, 46.7 (25.00) μg/mL in patients aged 19–65, and 38.5 (31.20) μg/mL in patients over 66 (*p* < 0.001, Kruskal–Wallis test).

### 2.4. Distribution of the Lithium and VPA Cp Samples in Relation to Gender

**Figure 6:**
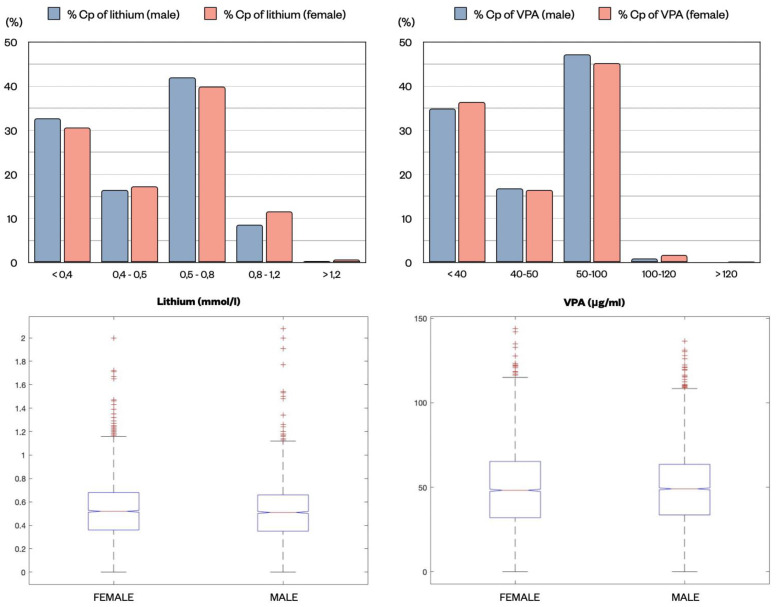
Distribution of lithium (mmol/L) and VPA (μg/mL) Cp expressed as percentage in the two subpopulations, males (blue) and females (pink), divided by different therapeutic ranges. No differences were found in the proportions (z-test). Lithium and VPA median concentrations for samples from males and females are reported in the lower graphs, showing highly similar values.

The distribution of Cp of the two drugs in males and females was analyzed to determine if any difference could be detected in these two subpopulations. A similar distribution was observed in both lithium and VPA Cp samples divided by ranges (z-test). Lithium median concentration in females was 0.52 mmol/L (IQR 0.32 mmol/L) and in males 0.51 mmol/L (0.31 mmol/L) (Figure 6). VPA median concentration was 48.25 μg/mL (33.35 μg/mL) in females vs. 49.1 μg/mL (30.00 μg/mL) in males. No statistically significant difference was found in the median values (Kruskal–Wallis test) (Figure 6).

### 2.5. Analysis of Multiple Dosing for Patients with Lithium and VPA Subtherapeutic Cp

Since a large number of samples was below the therapeutic range, we decided to inquire whether these values remained unchanged during the treatment or if they were modified in order to achieve the therapeutic window.

**Figure 7:**
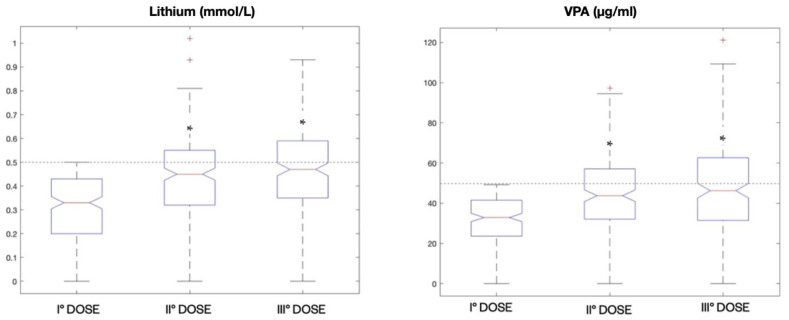
Boxplot representations of median lithium Cp (mmol/L) and VPA Cp (μg/mL) in patients (198) from the psychiatric unit receiving three consecutive dosing within the year starting from a subtherapeutic concentration. * *p* < 0.001 compared to the first dose by Kruskal–Wallis test.

By analyzing the whole sample of patients from the psychiatric unit, we found a total of 198 patients who were dosed for three consecutive times within the year starting with a lithium Cp < 0.5 mmol/L. These patients had a significant increase in the median Cp from the first dosing (0.33 mmol/L) to the second (0.45 mmol/L), but less from the second to the third (0.47 mmol/L) (*p* < 0.001, Kruskal–Wallis test). However, this increase did not allow for the therapeutic window in most patients to be reached (Figure 7).

A similar analysis was performed for VPA, and we found 180 patients with an initial Cp < 50 μg/mL who received three consecutive dosages within the year. The median VPA Cp was 32.9 μg/mL for the first dosage, 43.75 μg/mL for the second and 46.25 μg/mL for the third. The increase in VPA median levels was significant from the first one to the second, but not from the second to the third (*p* < 0.001, Kruskal–Wallis test); however, the therapeutic range was not reached (Figure 7).

### 2.6. Distribution of the Lithium and VPA Cp Samples in Combination Therapy

**Figure 8:**
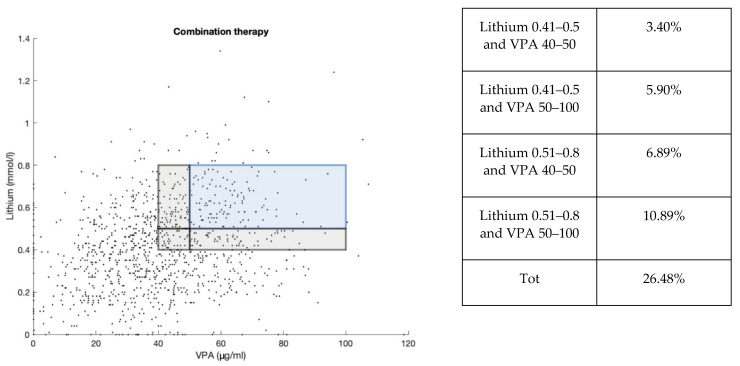
Scatter plot representing the distribution of lithium Cp (mmol/L) and VPA Cp (μg/mL) samples in combination therapy (1118). Samples in the therapeutic range of lithium (0.5–0.8 mmol/L) and VPA (50–100 μg/mL) are highlighted in the blue area. An additional gray area shows values of lithium (0.4–0.5 mmol/L) and VPA (40–50 μg/mL) close to the therapeutic range. These percentage values are reported in the table.

From our analysis, we found that 1118 samples were dosed for both lithium and VPA. Of these samples, only 10.29% were in the therapeutic window or 26.48% if we consider as lower limits 0.4 mmol/L for lithium and 40 μg/mL for VPA, an approach that could be considered legitimate when these two medications are given together (Figure 8). The scatter plot showed a large amount of samples at subtherapeutic Cp, as it was seen in lithium and VPA total data. In addition, 29.70% of samples in association were with lithium below 0.4 mmol/L and VPA 40 μg/mL, while 2.95% of cases were with lithium above 0.8 mmol/L and VPA above 100 μg/mL.

## 3. Discussion

TDM is a commonly used tool in clinical practice for neuropsychiatric disorders, and as pointed out by the recent guidelines, it is strongly recommended for several drugs [5]. In terms of cost effectiveness and cost benefits, preliminary studies have shown promising results, thus confirming the utility of this tool for different class of drugs such as lithium, antiepileptics and antidepressants [32]. Despite this evidence, TDM is still underused; for example, according to a recent report, during lithium therapy, 30–40% of patients did not receive drug plasma monitoring over a 12-month period [33].

Moreover, according to a recent survey report, only a small percentage of doctors reported to change the dose when the Cp was below the therapeutic range [7].

On this topic, in our study, we found that in several cases of patients receiving multiple blood sampling the initial sub-therapeutical Cp changed only partially without reaching the therapeutic window.

In addition, from our analysis, it has emerged that about 50% of the Cp of lithium and VPA is below the therapeutic range, of which about 32% of lithium is below 0.4 mmol/L and about 35% of VPA is below 40 μg/mL—values whose clinical efficacy has not been proven thus far. Other drug monitoring studies have also found that a relevant percentage of the Cp of lithium and VPA was below the therapeutic value [34]. For example, a retrospective analysis of TDM of 4359 samples of lithium in patients conducted over a period of 4 years (2000–2004) in India showed that lithium subtherapeutic levels were about 20% [35].

For different factors such as age and gender, we found that Cp values of lithium and VPA in the adolescents group were more in the therapeutic range compared to the other categories, while in the elderly group they were the lowest.

In relation to lithium clinical efficacy, a review on randomized clinical trials during 1966–2006 on long-term use has confirmed that the optimal response is between 0.6–0.75 mmol/L and that the minimum efficacious level was 0.4 mmol/L [22]. In other studies, it has been found that BD patients with a Cp of lithium below 0.5 mmol/L are associated with greater risk of recurrence of an episode and/or worsening of other symptoms [23,36].

Similar indications have been found for VPA in relation to its clinical efficacy compared to its Cp. The optimal therapeutic response can be achieved when Cp is between 75 and 99 μg/mL, and a linear correlation between VPA Cp and clinical efficacy was proposed [29], as it was found for lithium in the historical study of Amdisen et al. [37].

A possible explanation for our results could be that clinicians pay more attention to avoid side effects, especially in the long-term, with a tendency to keep low doses when possible. Alternatively, it is well known that polytherapy is often prescribed in psychiatric disorders where each single medication can be used at lower dosage. However, this approach in clinical practice is not completely backed by solid evidence, and data on the use of psychodrugs at doses lower than standard are scarce.

In relation to lithium, some in vitro studies have found that, at lower concentrations, it maintains neuroprotective activities against a wide variety of insults, including oxidative stress [38,39]. Conversely, in patients, the efficacy of lithium at lower doses lacks evidence, as only one study showed some metabolic and structural changes in the brain of psychotic patients [40].

For VPA, its activity at lower doses has not been studied systematically and evidence is lacking, besides one study carried out on cyclothymia and another one on Alzheimer’s patients [41,42].

From our analysis, it also emerged that VPA is extensively used to treat BDs, either alone or frequently in association with lithium. In some clinical conditions such as mixed states, rapid cycling, and BDs with comorbidities, lithium monotherapy is often not sufficient to improve most of the symptoms, and augmentation with VPA has generally resulted in a better outcome [16].

Lithium and VPA are the most used mood stabilizers in BDs, and despite sharing some common targets, they have different mechanisms of action [43]. The association between lithium and VPA is considered a good option, and it seems well tolerated in clinical practice [44,45].

In this prospective, this combination could allow, in theory, for a reduction of the dosages of both drugs in order to limit their risk of toxicity; however, this possibility also lacks solid scientific evidence. For example, there is no clear evidence that lithium plus VPA is better than lithium alone in preventing recurrence of episodes and in reducing hospitalization in BD-I, or that it could allow a reduction of dosage [46].

Surprisingly, there are no studies addressing this topic, and to the best of our knowledge, only one study has shown that when lithium and VPA are used in combination, the therapeutic range for lithium reduces to 0.4–0.8 mmol/L during chronic treatment [26]. However, more studies are required to confirm this possibility.

Our drug monitoring has confirmed the tendency of using lower dosages when lithium and VPA are used in association, where only 26% of the patients had a Cp of these two drugs in the therapeutic range, even by considering the lower limit of 0.4 mmol/L for lithium and 40 μg/mL for VPA.

While treating BDs, we should also mention that atypical antipsychotics could be used in association with lithium and VPA, especially in the acute phase of the disorder [18]. Thus, in our analysis, we should expect that, for some patients, atypical antipsychotics might also have been used in association with lithium or VPA, without being blood monitored.

In conclusion, from our present study, it emerged that a high percentage of patients under lithium and VPA treatment, either alone or in association, have sub-therapeutical Cp. Conversely, toxic levels of these drugs are practically never present.

However, limitations to our study include a lack of knowledge regarding the clinical information about the patients and the possibility of the use of other drugs in combination with lithium and VPA (e.g., antipsychotics, anxiolytics, etc.) that have not been monitored. This is an initial retrospective study based on a large number of laboratory data, which should be followed by a prospective clinical trial to correlate patient clinical response to lithium and VPA Cp.

## 4. Materials and Methods

### 4.1. Sample of the Study

Lithium and VPA blood samples for the period 2016–2020 were collected and analyzed at the Unit of Pharmacology and Pharmacogenetics of the Hospital of University of Pisa (Azienda Ospedaliero-Universitaria Pisana, AOUP). The total number of lithium samples was 7449, where 4275 came from the psychiatry unit, 864 came from the child neuropsychiatry unit and the remaining 2310 came from other units. The total number of VPA samples was 12,294, where 5087 (41.38%) came from psychiatry unit, 1640 (13.34%) came from child neuropsychiatry and the remaining 5567 (45.28%) came from other units. No exclusion criteria were applied to our data selection.

### 4.2. Analytical Determination of Lithium and VPA

Samples were collected following routinely clinical practice every morning from patients before taking the next dose (at steady state). Samples were stored in the refrigerator at 4 °C. Centrifugation and the analysis were carried out in serum for lithium and in plasma for VPA. Lithium Cp was measured using a colorimetric assay, while VPA Cp was measured with a chemiluminescent microparticle immunoassay (ARCHITECT).

The quantitative measurement of lithium was performed with a colorimetric determination, using Roche/Hitachi cobas c system. Lithium in the sample reacts with a porphyrin compound causing absorbance changes, which is directly proportional to the lithium concentration in the sample. For the detection of lithium, the patient’s sample was 5 µL of serum diluted in 100 µL of diluent (H_2_O). The measuring range was 0.05–3.00 mmol/L (0.03–2.08 mg/dL), with the lower limits of measurement as follows: limit of blank (LoB) = 0.03 mmol/L (0.02 mg/dL); limit of detection (LoD) = 0.05 mmol/L (0.03 mg/dL). The LoB and LoD were determined in accordance with the Clinical and Laboratory Standards Institute (CLSI) EP17-A requirements.

The quantitative measurement of VPA was performed with the ARCHITECT iValproic Acid assay, a chemiluminescent microparticle immunoassay. The analysis was performed on plasma samples. Plasma is combined with anti-valproic acid coated paramagnetic microparticles and valproic acid acridinium-labeled conjugate to create a reaction mixture. The anti-valproic acid coated microparticles bind to valproic acid present in the sample and to the valproic acid acridinium-labeled conjugate. The resulting chemiluminescent reaction is measured as relative light units. For the determination of valproic acid, 150 µL of plasma was used. The LoB and LoD of the ARCHITECT i Valproic Acid assay were determined according to CLSI Protocol EP17-A21, with a LoB = 0.27 μg/mL and LoD = 0.51 μg/mL.

The results were then registered in the Openlis software, a database accessed by the Hospital of the University of Pisa by using a card for recognition with password in order to protect the privacy of the patients. Our laboratory activity is ISO9001 certified, and it is routinely tested with external samples for quality control.

### 4.3. Data Analysis

The number of samples was reported for different subgroups (years, origin, range intervals, age). Proportional distribution of subgroups was compared using the z-test. Cp across different ages was reported as median, and Kruskal–Wallis test was performed. The level of significance was set at 0.05. The graphs and descriptive analysis were made with MATLAB and Statistics Toolbox Release 2021a (The MathWorks, Inc., Natick, MA, USA); z-test and Kruskal–Wallis tests were performed using SigmaPlot version 12.0 (Systat Software, Inc., Chicago, IL, USA).

## Figures and Tables

**Table 1:** The total number of samples collected yearly are reported for both lithium and VPA, together with Cp median values and the relative interquartile range (IQR).

	Lithium		VPA	
	Samples (n)	[Median] mmol/L (IQR)	Samples (n)	[Median] µg/mL (IQR)
2016	205	0.46 (0.26)	1829	47.40 (30.80)
2017	1529	0.50 (0.30)	2660	47.20 (31.25)
2018	2188	0.52 (0.31)	2908	49.50 (31.25)
2019	1989	0.52 (0.31)	2642	49.25 (32.20)
2020	1538	0.53 (0.33)	2255	50.20 (31.70)
Total	7449	0.52 (0.31)	12294	48.70 (31.60)

## Data Availability

Data is contained within the article.
